# Association of Interfacility Transfer and Patient and Hospital Characteristics With Thumb Replantation After Traumatic Amputation

**DOI:** 10.1001/jamanetworkopen.2020.36297

**Published:** 2021-02-03

**Authors:** Jessica I. Billig, Jacob S. Nasser, Hoyune E. Cho, Ching-Han Chou, Kevin C. Chung

**Affiliations:** 1 Section of Plastic Surgery, Department of Surgery, Michigan Medicine, Ann Arbor; 2 The George Washington University School of Medicine and Health Sciences, Washington, DC; 3 Department of Plastic Surgery, University of California, Irvine; 4 Center for Artificial Intelligence in Medicine, Chang Gung Memorial Hospital, Taoyuan, Taiwan

## Abstract

**Importance:**

Given that 40% of hand function is achieved with the thumb, replantation of traumatic thumb injuries is associated with substantial quality-of-life benefits. However, fewer replantations are being performed annually in the US, which has been associated with less surgical expertise and increased risk of future replantation failures. Thus, understanding how interfacility transfers and hospital characteristics are associated with outcomes warrants further investigation.

**Objective:**

To assess the association of interfacility transfer, patient characteristics, and hospital factors with thumb replantation attempts and success.

**Design, Setting, and Participants:**

This cross-sectional study used data from the US National Trauma Data Bank from 2009 to 2016 for adult patients with isolated traumatic thumb amputation injury who underwent revision amputation or replantation. Data analysis was performed from May 4, 2020, to July 20, 2020.

**Exposures:**

Interfacility transfer, defined as transfer of a patient from 1 hospital to another to obtain care for traumatic thumb amputation.

**Main Outcomes and Measures:**

Replantation attempt and replantation success, defined as having undergone a replantation without a subsequent revision amputation during the same hospitalization. Multilevel logistic regression models were used to assess the associations of interfacility transfer, patient characteristics, and hospital factors with replantation outcomes.

**Results:**

Of 3670 patients included in this analysis, 3307 (90.1%) were male and 2713 (73.9%) were White; the mean (SD) age was 45.8 (16.5) years. A total of 1881 patients (51.2%) were transferred to another hospital; most of these patients were male (1720 [91.4%]) and White (1420 [75.5%]). After controlling for patient and hospital characteristics, uninsured patients were less likely to have thumb replantation attempted (odds ratio [OR], 0.61; 95% CI, 0.47-0.78) or a successful replantation (OR, 0.64; 95% CI, 0.49-0.84). Interfacility transfer was associated with increased odds of replantation attempt (OR, 1.34; 95% CI, 1.13-1.59), with 13% of the variation at the hospital level. Interfacility transfer was also associated with increased replantation success (OR, 1.23; 95% CI, 1.03-1.47), with 14% of variation at the hospital level.

**Conclusions and Relevance:**

In this cross-sectional study, interfacility transfer and particularly hospital-level variation were associated with increased thumb replantation attempts and successes. These findings suggest a need for creating policies that incentivize hospitals with replantation expertise to provide treatment for traumatic thumb amputations, including promotion of centralization of replantation care.

## Key Points

**Question:**

What patient- and hospital-level factors are associated with thumb replantation outcomes after traumatic thumb amputation?

**Findings:**

In this cross-sectional study of 3670 patients, the rates of thumb replantation attempts and success were higher among patients who were transferred to another facility. Hospital characteristics were associated with substantial variation in replantation outcomes, and patient characteristics, including insurance status, were associated with fewer thumb replantation attempts.

**Meaning:**

These findings suggest that minimizing between-hospital differences in the provision of replantation care may be associated with improved quality, equitability, and delivery of such care.

## Teaser

This cross-sectional study examines associations of interfacility transfer and patient and hospital characteristics with thumb replantation attempts and success after traumatic thumb amputation.

## Introduction

Hand injuries account for 12% of all traumatic injuries in the US, many of which consist of traumatic digit amputations.^1^ Traumatic thumb amputations are associated with social concerns for the patient, functional decline, and considerable economic consequences for the community attributed to patient absenteeism and loss of employment.^2,3^ Recent evidence suggests that digit replantation may be associated with substantial benefit compared with revision amputation owing to improved function and appearance of the hand.^4,5,6^ In a recent study, patients with thumb amputations who underwent replantation reported better outcomes compared with patients who underwent revision amputation; thus, thumb amputation has been considered an absolute indication for replantation.^5^ Despite the added benefits associated with replantation, there has been a decreasing trend in the rates of replantation attempts and subsequent success.^7,8,9^ In the US, replantation success rates have decreased from approximately 75% in the early 2000s to approximately 66% from 2010 to 2012.^8^ In contrast, in countries such as China and Japan replantation success rates are approximately 90%.^10^

Replantation requires complex microsurgical expertise, which is not available at all facilities; therefore, interfacility transfer is commonly required. Interfacility transfer may be associated with delays in care and thus with overall patient outcomes. However, the effect of interfacility transfer on replantation attempt and success among patients with a traumatic thumb amputation remains unknown. For other traumatic conditions, interfacility transfer is a common occurrence. In a study of pediatric trauma injuries, female sex, race, and lack of insurance were associated with interfacility transfer, resulting in delays to definitive treatment.^11^ However, Nathens et al^12^ evaluated transfers from lower-level trauma centers to higher-level trauma centers and found that interfacility transfers were not associated with mortality rate, length of stay, or hospital charges. Given the complex nature of replantation, transfer to a higher-level trauma center with more resources and more experienced surgeons may provide a benefit to the patient; however, the timeliness of the transfer may be associated with additional challenges for replantation. Identifying the effect of interfacility transfer on replantation outcomes may influence policy focused on centralization of care for patients undergoing traumatic digit amputation.

In this population-based cross-sectional study, we performed an analysis of adult patients with isolated traumatic thumb amputation injury who underwent revision amputation or replantation to investigate the association of interfacility transfers with replantation attempts and success. In addition, we assessed the patient and hospital characteristics associated with these replantation outcomes.

## Methods

### Data Source and Cohort Selection

We used data from the National Trauma Data Bank (NTDB) from 2009 to 2016 to conduct a retrospective cross-sectional study. The NTDB is the largest aggregated trauma registry in the US; it contains more than 7.5 million records^13^ collected from more than 900 different trauma centers within the US.^14^ The NTDB contains patient-level health care data including emergency department visits and inpatient encounters. The institutional review board at the University of Michigan designated this study not regulated, and the need for informed consent was waived. This study followed the Strengthening the Reporting of Observational Studies in Epidemiology (STROBE) reporting guideline.

From the NTDB, we examined data for patients 18 years or older with an isolated traumatic thumb amputation who underwent either revision amputation or replantation. Eligibility was determined using *International Classification of Diseases, Ninth Revision *(*ICD-9*) and *International Classification of Diseases and Related Health Problems, Tenth Revision* (*ICD-10*) diagnosis and procedure codes (eTable 1 in the Supplement). We included only patients who had a traumatic thumb amputation and excluded those with other injuries on other digits. We excluded patients if they were younger than 18 years or if they were pronounced dead on arrival or died in the emergency department (eFigure in the Supplement).

### Variables of Interest

Our primary outcome variables were replantation attempt and replantation success. Replantation attempt was defined using the *ICD-9* or *ICD-10* procedure code for replantation during the primary hospitalization (eTable 1 in the Supplement). Replantation success was defined as replantation without a subsequent revision amputation during the primary hospitalization.

For independent variables, we included patient-level and hospital-level characteristics. Patient-level factors included age, sex, race/ethnicity, insurance status, and presence of comorbidities available in the NTDB. For insurance status, *self-pay* was used to denote lack of insurance. Comorbidities included hypertension, current smoker, diabetes, chronic obstructive pulmonary disease, alcohol use disorder, bleeding disorders, congestive heart failure, cerebrovascular accident, myocardial infarction, functionally dependent status, disseminated cancer, steroid use, angina pectoris, and current chemotherapy.^15^ For injury-related characteristics, we used the Injury Severity Score (ISS) to quantify the severity of the trauma. An ISS greater than 15 denotes a major trauma or multiple traumas.^16^ In addition, we determined whether the traumatic thumb amputation was attributable to a degloving or crush injury using the Abbreviated Injury Scale available in the NTDB. The Abbreviated Injury Scale is an injury score based on severity, type, and anatomic region of the injury.^17^ We also determined whether the patient was transferred to another facility, which is a variable available in the NTDB. We included hospital characteristics, such as hospital bed size, teaching status, and trauma center designation.

### Statistical Analyses

Data were analyzed from May 4, 2020, to July 20, 2020. We used descriptive statistics to assess unadjusted comparisons between patients who were transferred to another facility and those who were not. In addition, we compared the characteristics of hospitals that transferred patients to another facility and hospitals that did not transfer patients. We used a 2-tailed *t* test for continuous variables and the χ^2^ or Fisher exact test for categorical variables. We made unadjusted comparisons for replantation attempts and replantation success between patients who were transferred to another facility and those who did not using a 2-tailed *t* test.

Multilevel logistic regression models were used to examine the association among patient and hospital characteristics and the outcomes replantation attempt and replantation success. Given the clustering of patients within hospitals, we used random intercepts at the hospital level and fixed slopes. The models were adjusted for age, sex, race/ethnicity, insurance status, crush injury, degloving injury, ISS, interhospital transfer, trauma center designation, hospital teaching status, and bed size. Significance was set at *P* < .05 for all analyses. All analyses were performed using SAS software, version 9.4 (SAS Institute Inc).

## Results

We identified 3670 patients who sustained a traumatic thumb amputation from 2009 to 2016, of whom 3307 (90.1%) were male and 2713 (73.9%) were White; the mean (SD) age was 45.8 (16.5) years. A total of 1881 patients (51.2%) were transferred to another hospital; most of these patients were male (1720 [91.4%]) and White (1420 [75.5%]). There were no differences in age between patients who were transferred and those who were not transferred (mean [SD] age, 45.8 [16.5] years vs 45.9 [16.5] years; *P* = .96). A total of 346 patients (18.4%) who were transferred to another facility did not have insurance compared with 307 patients (17.2%) who were not transferred (*P* = .04). The median ISS of patients who were not transferred was 4 (interquartile range, 4-4) compared with 4 (interquartile range, 4-5) for patients who were transferred (*P* < .001). However, both groups had ISSs below 15, which is the cutoff to denote a major trauma or multiple traumas (Table 1).

**Table 1.** Patient Sociodemographic and Clinical Characteristics Stratified by Interfacility Transfer From 2009 to 2016^a^

Characteristic	Total cohort (N = 3670)	Patients transferred to another hospital (n = 1881)	Patients not transferred to another hospital (n = 1789)	*P* value^b^
Age, mean (SD), y	45.8 (16.5)	45.8 (16.6)	45.9 (16.5)	.96
Sex				
Male	3307 (90.1)	1720 (91.4)	1587 (88.7)	.01
Female	363 (9.9)	161 (8.6)	202 (11.3)	
Race				
White	2713 (73.9)	1420 (75.5)	1293 (72.3)	<.001
Black	236 (6.4)	90 (4.8)	146 (8.2)	
Other	721 (19.6)	371 (19.7)	350 (19.6)	
Hispanic or Latino ethnicity	600 (16.3)	298 (15.8)	302 (16.9)	.40
Insurance type				
Private	1101 (30.0)	562 (29.9)	539 (30.1)	.04
Medicare	450 (12.3)	235 (12.5)	215 (12.0)	
Medicaid	255 (6.9)	114 (6.1)	141 (7.9)	
Self-pay	653 (17.8)	346 (18.4)	307 (17.2)	
Other government	76 (2.1)	46 (2.4)	30 (1.7)	
Other, including workers’ compensation	938 (25.6)	474 (25.2)	464 (25.9)	
Unknown	197 (5.4)	104 (5.5)	93 (5.2)	
Comorbidity				
Hypertension	745 (20.3)	388 (20.6)	357 (20.0)	.61
Current smoker	782 (21.3)	420 (22.3)	362 (20.2)	.12
Diabetes	253 (6.9)	137 (7.3)	116 (6.5)	.34
Chronic obstructive pulmonary disease	131 (3.6)	70 (3.7)	61 (3.4)	.61
Alcohol use disorder	146 (4.0)	77 (4.1)	69 (3.9)	.71
Bleeding disorder	75 (2.0)	39 (2.1)	36 (2.0)	.90
Congestive heart failure	21 (0.6)	11 (0.6)	10 (0.6)	.92
Cerebrovascular accident	14 (0.4)	8 (0.4)	6 (0.3)	.66
Myocardial infarction	36 (1.0)	20 (1.1)	16 (0.9)	.60
Functionally dependent health status	7 (0.2)	1 (0.1)	6 (0.3)	.051
Disseminated cancer	8 (0.2)	3 (0.2)	5 (0.3)	.44
Steroid use	5 (0.1)	2 (0.1)	3 (0.2)	.61
Angina pectoris	4 (0.1)	2 (0.1)	2 (0.1)	.96
Currently receiving chemotherapy	5 (0.1)	2 (0.1)	3 (0.2)	.61
Crush injury	73 (2.0)	37 (2.0)	36 (2.0)	.92
Degloving injury	15 (0.4)	5 (0.3)	10 (0.6)	.16
Injury Severity Score				
Mean (SD)	5.1 (3.5)	4.7 (2.2)	5.4 (4.5)	<.001
Median (IQR)	4 (4-5)	4 (4-4)	4 (4-5)	<.001
Replantation				
Attempt	1063 (29.0)	638 (33.9)	425 (23.8)	<.001
Failure	214 (20.1)	136 (21.3)	78 (18.4)	.24

Abbreviation: IQR, interquartile range.

^a^ Data are presented as number (percentage) of patients unless otherwise indicated.

^b^ Bivariate comparisons were obtained using the χ^2^ test and Fisher exact test for categorical variables and a 2-tailed *t* test for continuous variables.

Compared with hospitals that did not transfer patients, hospitals that initiated interfacility transfers were larger (bed size >600) (90 [36.6%] vs 31 [15.4%]; *P* < .001), were more likely to be teaching hospitals (110 [44.7%] vs 38 [18.8%]; *P* < .001), and were more likely to have a level I trauma center designation (45 [18.3%] vs 17 [8.4%]; *P* < .001) (eTable 2 in the Supplement). In addition, hospitals that received transfers employed more orthopedic surgeons (>15 orthopedic surgeons), which serves as a proxy for surgical capacity (39 [15.9%] vs 24 [11.9%]; *P* < .001).

**Figure 1.**
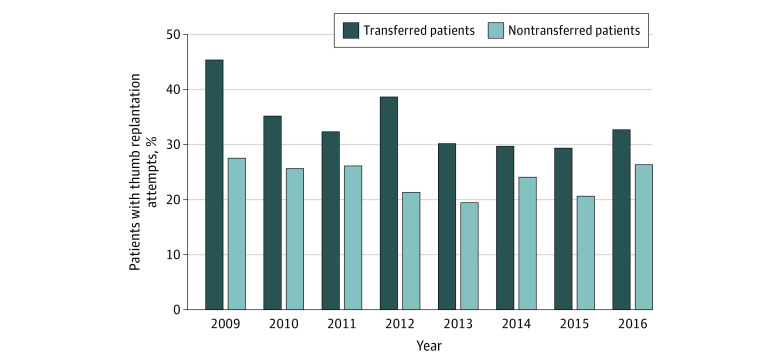
Thumb Replantation Attempts Among Patients Who Were and Were Not Transferred to Another Hospital From 2009 to 2016

**Figure 2.**
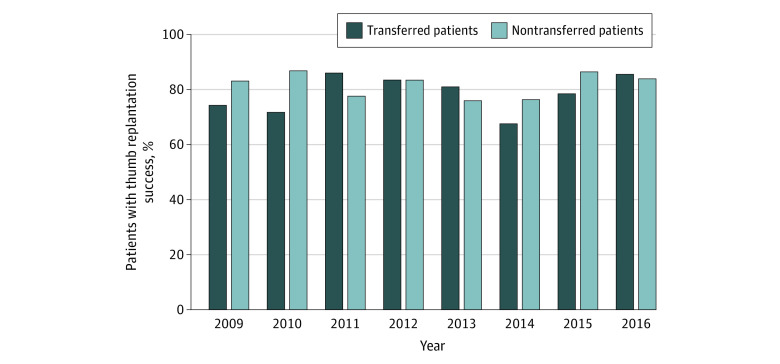
Thumb Replantation Success Among Patients Who Were and Were Not Transferred to Another Hospital From 2009 to 2016

Figure 1 shows the replantation attempts stratified by interfacility transfer over time. Unadjusted comparisons revealed that patients transferred to another facility were more likely to have a replantation attempt (unadjusted comparison: 34% [95% CI, 32%-36%] vs 24% [95% CI, 22%-26%]; *P* < .001) (Table 1). However, the success rate among patients who underwent replantation after transfer was not significantly different from the success rate among patients who were not transferred (unadjusted comparison, 21% [95% CI, 18%-25%] vs 18% [95% CI, 15%-22%]; *P* = .24) (Table 1). Figure 2 shows replantation success stratified by interfacility transfer over time.

After controlling for patient and hospital characteristics, older patients had lower odds of attempted replantation, with a 1% decrease in each additional year (odds ratio [OR], 0.99; 95% CI, 0.98-0.99; *P* < .001) (Table 2). Similarly, patients with Medicare had lower odds of replantation attempt (OR, 0.64; 95% CI, 0.46-0.88; *P* = .01). Patients who were uninsured (ie, self-pay) were less likely to have a replantation attempted (OR, 0.61; 95% CI, 0.47-0.78; *P* < .001). Patients who were transferred had greater odds of undergoing replantation attempt (OR, 1.34; 95% CI, 1.13-1.59; *P* < .001). Compared with university-associated hospitals, nonteaching hospitals and community hospitals were associated with decreased odds of attempted replantation (nonteaching: OR, 0.43 [95% CI, 0.27-0.69], *P* < .001; community: OR, 0.60 [95% CI, 0.44-0.83], *P* = .002) (Table 2). Trauma center level was not significantly associated with the odds of replantation attempt. However, 13% of the variation in attempts at replantation was found at the hospital level.

**Table 2.** Multilevel Logistic Regression of Factors Associated With Replantation Attempt for Traumatic Thumb Amputation

Covariate	OR (95% CI)	*P* value
Sex		
Female	0.93 (0.70-1.22)	.58
Male	1 [Reference]	NA
Age	0.99 (0.98-0.99)	<.001
Race		
White	1 [Reference]	NA
Black	0.77 (0.55-1.10)	.15
Asian	0.87 (0.42-1.79)	.70
Other	1.42 (1.04-1.94)	.03
Hispanic or Latino ethnicity	0.73 (0.54-0.99)	.04
Insurance status		
Private	1 [Reference]	NA
Medicaid	0.84 (0.60-1.17)	.30
Medicare	0.64 (0.46-0.88)	.007
Self-pay	0.61 (0.47-0.78)	<.001
Other government	0.68 (0.38-1.24)	.20
Other, including workers’ compensation	1.01 (0.82-1.24)	.95
Current smoker	0.89 (0.73-1.08)	.23
Crush injury	0.51 (0.27-0.96)	.04
Degloving injury	0.35 (0.07-1.61)	.18
ISS	0.90 (0.87-0.93)	<.001
Interfacility transfer		
No	1 [Reference]	NA
Yes	1.34 (1.13-1.59)	<.001
Trauma center ACS certification level		
I	1 [Reference]	NA
II	1.09 (0.74-1.61)	.65
III	1.01 (0.33-3.07)	.99
Teaching status		
University	1 [Reference]	NA
Nonteaching	0.43 (0.27-0.69)	<.001
Community	0.60 (0.44-0.83)	.002
Bed size		
≤200	0.86 (0.47-1.57)	.58
201-400	0.58 (0.40-0.84)	.005
401-600	0.85 (0.63-1.15)	.30
>600	1 [Reference]	NA

Abbreviations: ACS, American College of Surgeons; ISS, Injury Severity Score; NA, not applicable; OR, odds ratio.

In the analysis of replantation success, older patients had lower odds of success, with a 1% decrease in success for each additional year (OR, 0.99; 95% CI, 0.98-1.00; *P* < .001). Similarly, Medicare and uninsured patients were less likely to have a successful replantation (Table 3). Patients who were transferred had greater odds of replantation success (OR, 1.23; 95% CI, 1.03-1.47; *P* = .03). Compared with university-associated hospitals, nonteaching hospitals and community hospitals were associated with decreased odds of replantation success (nonteaching: OR, 0.49 [95% CI, 0.29-0.81], *P* = .006; community: OR, 0.64 [95% CI, 0.45-0.89], *P* = .009) (Table 3). Fourteen percent of the variation in replantation success was found at the hospital level.

**Table 3.** Multilevel Logistic Regression of Factors Associated With Replantation Success for Traumatic Thumb Amputation

Covariate	OR (95% CI)	*P* value
Sex		
Female	0.86 (0.64-1.16)	.34
Male	1 [Reference]	NA
Age	0.99 (0.98-1.00)	<.001
Race		
White	1 [Reference]	NA
Black	0.86 (0.59-1.25)	.43
Asian	1.04 (0.50-2.18)	.91
Other	1.38 (0.99-1.93)	.06
Hispanic or Latino ethnicity	0.74 (0.54-1.01)	.06
Insurance status		
Private	1 [Reference]	NA
Medicaid	0.80 (0.56-1.14)	.22
Medicare	0.69 (0.49-0.98)	.04
Self-pay	0.64 (0.49-0.84)	.001
Other government	0.88 (0.48-1.60)	.67
Other, including workers’ compensation	0.93 (0.75-1.17)	.55
Current smoker		.11
Crush injury	0.60 (0.31-1.17)	.13
Degloving injury	0.45 (0.10-2.11)	.31
ISS	0.90 (0.87-0.94)	<.001
Interfacility transfer		
No	1 [Reference]	NA
Yes	1.23 (1.03-1.47)	.03
Trauma center ACS certification level		
I	1 [Reference]	NA
II	0.93 (0.61-1.42)	.75
III	1.36 (0.46-4.04)	.58
Teaching status		
University	1 [Reference]	NA
Nonteaching	0.49 (0.29-0.81)	.006
Community	0.64 (0.45-0.89)	.009
Bed size		
≤200	0.88 (0.46-1.68)	.69
201-400	0.67 (0.45-1.00)	.054
401-600	0.92 (0.67-1.26)	.59
>600	1 [Reference]	NA

Abbreviations: ACS, American College of Surgeons; ISS, Injury Severity Score; NA, not applicable; OR, odds ratio.

## Discussion

In this cross-sectional study, we found that the rates of thumb replantation attempt and success were higher for patients who were transferred to another facility. Hospital characteristics were associated with substantial variation in replantation outcomes, suggesting that minimizing between-hospital differences in the provision of replantation care to improve quality and delivery of care in an equitable manner is needed. In addition, uninsured patients included in the sample were less likely to have a replantation attempt or experience replantation success, which represents a patient population that may be receiving suboptimal care. Thus, policy aimed at improving the outcomes for patients with traumatic thumb amputation should include consideration of centralizing replantation care.

Despite evidence suggesting that replantation is associated with benefits in function, emotional well-being, and economic productivity, the rates of replantation continue to decrease across the US.^7,8,9,18^ This decrease in replantation rates is especially concerning for patients who have sustained traumatic thumb amputation because 40% of hand function is achieved with the thumb.^19,20^ In an analysis of the National Electronic Injury Surveillance System, Bureau of Labor Statistics, and National Inpatient Sample databases, the number of digit replantations decreased by more than 50% between 2001 and 2011 in spite of a stable incidence of traumatic digit amputations.^18^ Moreover, a population-based analysis showed decreases in the rate of replantation attempts regardless of a hospital’s geographic location.^7^ This decrease in replantation attempts has been associated with a decrease in replantation success in the US, likely because of decreased surgical expertise.^8^ In our analysis of the data from 2009 to 2016 in the NTDB, we found a similar result: the rate of replantation attempts decreased while the rate of revision amputations increased. The decreasing rate of replantation is associated with multiple factors, including increasing costs of health care services, limited availability of hand surgeons with microsurgical expertise, staffing issues, and low insurance reimbursement.^1,8,21^ However, in other developed countries including Taiwan, Japan, and China, rates of replantation and success rates are higher than in the US.^10,22,23,24^ Specialized replantation centers in these countries that streamline replantation care may contribute to the replantation success. Therefore, establishing centers of excellence for digit replantation in the US may help mitigate this decrease in replantation through concentration of case volume, referral network development, and streamlined delivery of replantation care through specialization.

Centralization of care for complex medical conditions has been identified as an avenue to improve outcomes and reduce costs in various surgical fields.^25,26,27,28^ Sakai-Bizmark et al^26^ conducted a population-based analysis of pediatric cardiac surgical procedures and found that centralization was associated with reductions in mortality, morbidity, and cost. Moreover, Warner et al^25^ conducted a retrospective analysis of patients with ruptured abdominal aortic aneurysm to evaluate the association of centralized care with outcomes and found that the mortality rate was decreased by approximately 20% in association with increased technology and availability of vascular surgeons. We found that the odds of replantation attempt and success were greater among patients who were transferred to another facility. The hospitals that attempted replantation included teaching hospitals, level 1 trauma centers, and those with a larger number of hospital beds and orthopedic surgeons, reinforcing the notion that health care systems with specialized surgeons and expertise in replantation may provide better replantation care. However, hospital-level variation in trauma care in the US continues to be associated with poor clinical outcomes and increased costs.^29,30,31,32,33^ Because of the cross-sectional nature of this study, we are unable to assess longer-term replantation success (ie, after the index hospitalization), which may shed light on the nationwide variation in replantation care. In a population-level analysis of traumatic thumb amputations, Mahmoudi et al^32^ found that teaching hospitals, hospitals with a high volume of patients with traumatic injuries, and level 1 trauma centers were more likely to attempt replantation. Our study corroborates these findings, with 13% of variation in replantation attempts and 14% of variation in replantation success found at the hospital level, underscoring the association between-hospital differences and replantation outcomes. The American College of Surgeons and the American Society for Surgery of the Hand have jointly made substantial efforts to centralize hand trauma care. A pilot program has been initiated to designate centers that have the ability to offer the full spectrum of hand surgical care at all hours.^34^ Currently, financial backing is limited, and policies are needed to incentivize both physicians and hospital systems. These efforts provide the framework for establishing centralized hand surgery centers with microsurgical expertise. Therefore, our findings suggest that to improve replantation outcomes, policies to centralize replantation care and establish financial incentives are needed to create streamlined systems-level processes to provide surgical expertise and efficient care.

Research has shown that lack of health insurance is negatively associated with outcomes, contributing to widespread health care disparities.^35,36,37,38^ Specifically, in a cross-sectional analysis of the National Emergency Department Sample, Venkatesh et al^36^ found that patients without insurance or patients with Medicaid insurance received a different level of hospital care compared with privately insured patients. In addition, Connolly et al^38^ performed an analysis of surgical outcomes after coronary artery bypass grafting and found that patients with Medicaid insurance experienced significantly worse outcomes compared with those with private insurance. Our analysis of patients with traumatic thumb amputation corroborates these findings, revealing that uninsured patients were less likely to have a replantation attempted even after controlling for both patient and hospital characteristics. Moreover, among patients with traumatic thumb amputation who underwent replantation, the odds of success were lower for those without insurance compared with those with private insurance. This finding may be related to the higher cost of care associated with replantation compared with revision amputation, and it is not known whether these findings are associated with factors related to the patient, surgeon, or hospital. Nonetheless, if variation in care is reduced through the development of specialized replantation centers, hospitals may have more incentives to offer thumb replantation regardless of insurance status. In addition, policies that focus on reducing disparities in outcomes for patients with traumatic thumb amputation with different insurance status appear to be needed. Such policies can be established by means of additional hospital quality metrics and certification initiatives aimed at improving access to equitable hand surgical care.^36^

### Limitations

This study has limitations. The NTDB is subject to selection bias because data collection is voluntary. Because higher-performing hospitals are more likely to contribute to the database, our findings may be overestimates of the rate of attempted replantation and successful procedures. However, we would expect that transferred patients are more likely to have sustained complex injuries, thus contributing to the selection bias. We would expect that these injury patterns would be associated with a lower success rate of replantation. However, we found that interfacility transfer was associated with increased odds of replantation success. Also, the database does not include data from patients who die before the transfer takes place, which may also contribute to the possible overestimation.^39^ Nonetheless, patients with isolated traumatic thumb amputation do not commonly die before they receive treatment. Furthermore, surgeon-level variation may account for the hospital-level variation found in this study; however, the database does not include data on surgical expertise such as an individual surgeon’s case volume. Because the NTDB data consist of initial emergency department visits and subsequent hospitalizations, we could not assess the rate of long-term success associated with attempted replantation, which may also vary at the hospital level.

## Conclusions

In this cross-sectional study, interfacility transfer was associated with increased thumb replantation attempts and successes. However, there was substantial hospital-level variation in replantation outcomes. These findings suggest a need for creating policies that incentivize hospitals with replantation expertise to provide treatment for traumatic thumb amputations, including promotion of centralization of replantation care.

## Notes

Supplement. **eTable 1.** Diagnostic and Procedural Codes for Traumatic Thumb Amputation and Treatment **eFigure.** Cohort Flow Diagram **eTable 2.** Hospital Characteristics (2009-2016)
