# Supplementary material for: Association of Interfacility Transfer and Patient and Hospital Characteristics With Thumb Replantation After Traumatic Amputation
Source: JAMA Netw Open. 2021 Feb 3;4(2):e2036297. doi: 10.1001/jamanetworkopen.2020.36297 (PMC7859845; doi:10.1001/jamanetworkopen.2020.36297)
Supplement: Supplement. — eTable 1. Diagnostic and Procedural Codes for Traumatic Thumb Amputation and Treatment eFigure. Cohort Flow Diagram eTable 2. Hospital Characteristics (2009-2016) [file jamanetwopen-e2036297-s001.pdf]

Supplementary Online Content

Billig JI, Nasser JS, Cho HE, Chou CH, Chung KC. Association of interfacility transfer and patient and hospital characteristics with thumb replantation after traumatic amputation. *JAMA Netw Open*. 2021;4(2):e2036297.
doi:10.1001/jamanetworkopen.2020.36297

eTable 1. Diagnostic and Procedural Codes for Traumatic Thumb Amputation and Treatment

eFigure. Cohort Flow Diagram

eTable 2. Hospital Characteristics (2009-2016)

This supplementary material has been provided by the authors to give readers additional information about their work.

© 2021 Billig JI et al. *JAMA Network Open*.

eTable 1. Diagnostic and Procedural Codes for Traumatic Thumb Amputation and Treatment

Category	Code	Description
Diagnosis Codes		
Traumatic Thumb Amputation (ICD-9 Diagnosis Codes)	885.0	Traumatic amputation of thumb
	885.1	Traumatic amputation of thumb, complicated
Traumatic Thumb Amputation (ICD-10 Diagnosis Codes)	S68.011A	Complete traumatic metacarpophalangeal amputation of right thumb, initial encounter
	S68.012A	Complete traumatic metacarpophalangeal amputation of left thumb, initial encounter
	S68.019A	Complete traumatic metacarpophalangeal amputation of unspecified thumb, initial encounter
	S68.021A	Partial traumatic metacarpophalangeal amputation of right thumb, initial encounter
	S68.022A	Partial traumatic metacarpophalangeal amputation of left thumb, initial encounter
	S68.029A	Partial traumatic metacarpophalangeal amputation of unspecified thumb, initial encounter
	S68.511A	Complete traumatic transphalangeal amputation of right thumb, initial encounter
	S68.512A	Complete traumatic transphalangeal amputation of left thumb, initial encounter
	S68.519A	Complete traumatic transphalangeal amputation of unspecified thumb, initial encounter
	S68.521A	Partial traumatic transphalangeal amputation of right thumb, initial encounter
	S68.522A	Partial traumatic transphalangeal amputation of left thumb, initial encounter
	S68.529A	Partial traumatic transphalangeal amputation of unspecified thumb, initial encounter
Procedural Codes		
Replantation (ICD-9 Procedure Codes)	84.21	Thumb reattachment
Replantation (ICD-10 Procedure Codes)	0XML0ZZ	Reattachment of Right Thumb, Open Approach
	0XMM0ZZ	Reattachment of Left thumb, open approach
Revision Amputation (ICD-9 Procedure Codes)	84.02	Amputation and disarticulation of thumb
	84.3	Revision of amputation stump
Revision Amputation (ICD-10 Procedure Codes)	0X6J0Z4	Detachment at Right Hand, Complete 1st Ray, Open Approach
	0X6J0Z9	Detachment at Right Hand, Partial 1st Ray, Open Approach

© 2021 Billig JI et al. *JAMA Network Open*.

	0X6K0Z4	Detachment at Left Hand, Complete 1st Ray, Open Approach
	0X6K0Z9	Detachment at Left Hand, Partial 1st Ray, Open Approach
	0X6L0Z0	Detachment of Right thumb, complete, open approach
	0X6L0Z1	Detachment of Right thumb, high, open approach
	0X6L0Z2	Detachment of Right thumb, mid, open approach
	0X6L0Z3	Detachment of Right thumb, low, open approach
	0X6M0Z0	Detachment of Left thumb, complete, open approach
	0X6M0Z1	Detachment of Left thumb, high, open approach
	0X6M0Z2	Detachment of Left thumb, mid, open approach
	0X6M0Z3	Detachment of Left thumb, low, open approach

© 2021 Billig JI et al. *JAMA Network Open*.

eFigure 1. Cohort Flow Diagram

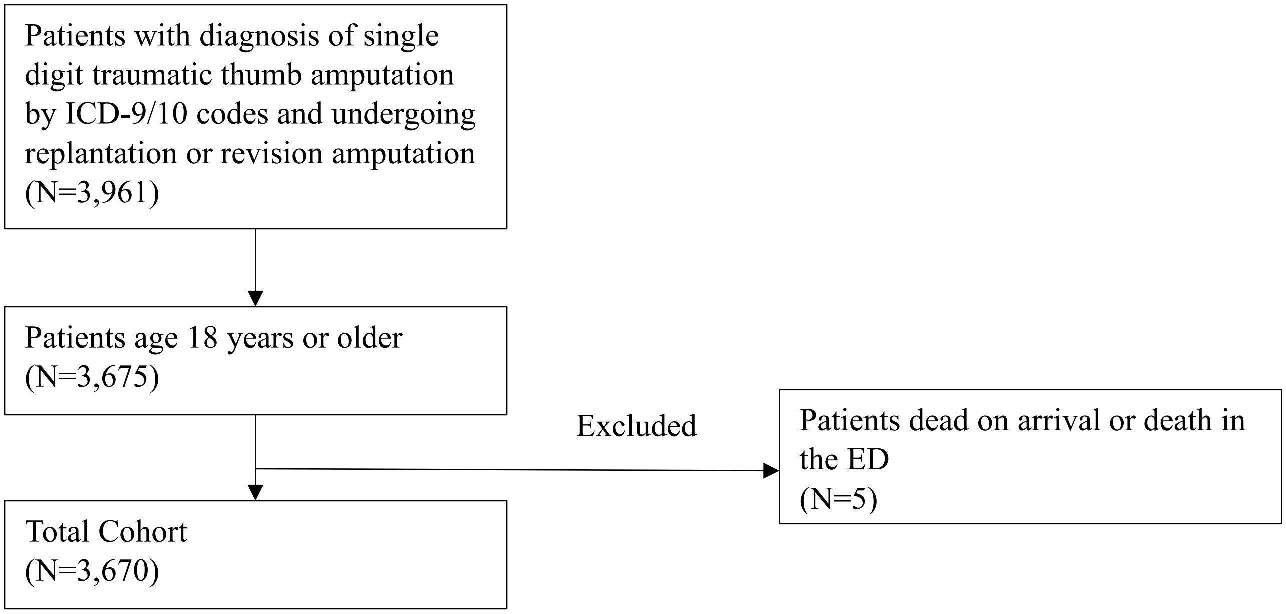

© 2021 Billig JI et al. *JAMA Network Open*.

eTable 2. Hospital Characteristics

	Hospitals That Received Transfer (N=246)	Hospitals That Did Not Transfer (N=202)	P-Value[a]
Bed Size			<0.001
≤ 200	15 (6.1)	30 (14.9)	
201-400	73 (30.0)	86 (42.6)	
401-600	68 (27.6)	53 (26.2)	
>600	90 (36.6)	31 (15.3)	
Unknown	0 (0)	2 (1.0)	
Bed Size[b]			<0.001
≤600	156 (63.4)	171 (84.7)	
>600	90 (36.6)	31 (15.4)	
Teaching Status			<0.001
University	110 (44.7)	38 (18.8)	
Non-teaching	41 (16.7)	65 (32.2)	
Community	95 (38.6)	97 (48.0)	
Unknown	0 (0)	2 (1.0)	
Trauma Center ACS Verification			<0.001
I	45 (18.3)	17 (8.4)	
II	40 (16.2)	48 (23.8)	
III	3 (1.2)	14 (6.9)	
Other	158 (64.2)	123 (60.9)	
Trauma Center ACS Verification[b]			0.003
I	45 (18.3)	17 (8.4)	
II, III, and Other	201 (81.7)	185 (91.6)	
Number of Orthopaedic Surgeons			<0.001
1-4	28 (11.4)	49 (24.3)	
5-6	46 (18.7)	34 (16.8)	
7-9	48 (19.5)	51 (25.2)	
10-15	85 (34.6)	44 (21.8)	
>15	39 (15.9)	24 (11.9)	
Number of Trauma Surgeons			0.10
0	1 (0.4)	0 (0)	
1-3	12 (4.9)	22 (10.9)	
4-6	142 (57.7)	113 (55.9)	
7-8	44 (17.9)	38 (18.8)	
>8	47 (19.1)	29 (14.4)	

[a]Bivariate comparisons obtained by Chi-square test.
[b]Variable collapsed into binary variable.

© 2021 Billig JI et al. *JAMA Network Open*.
